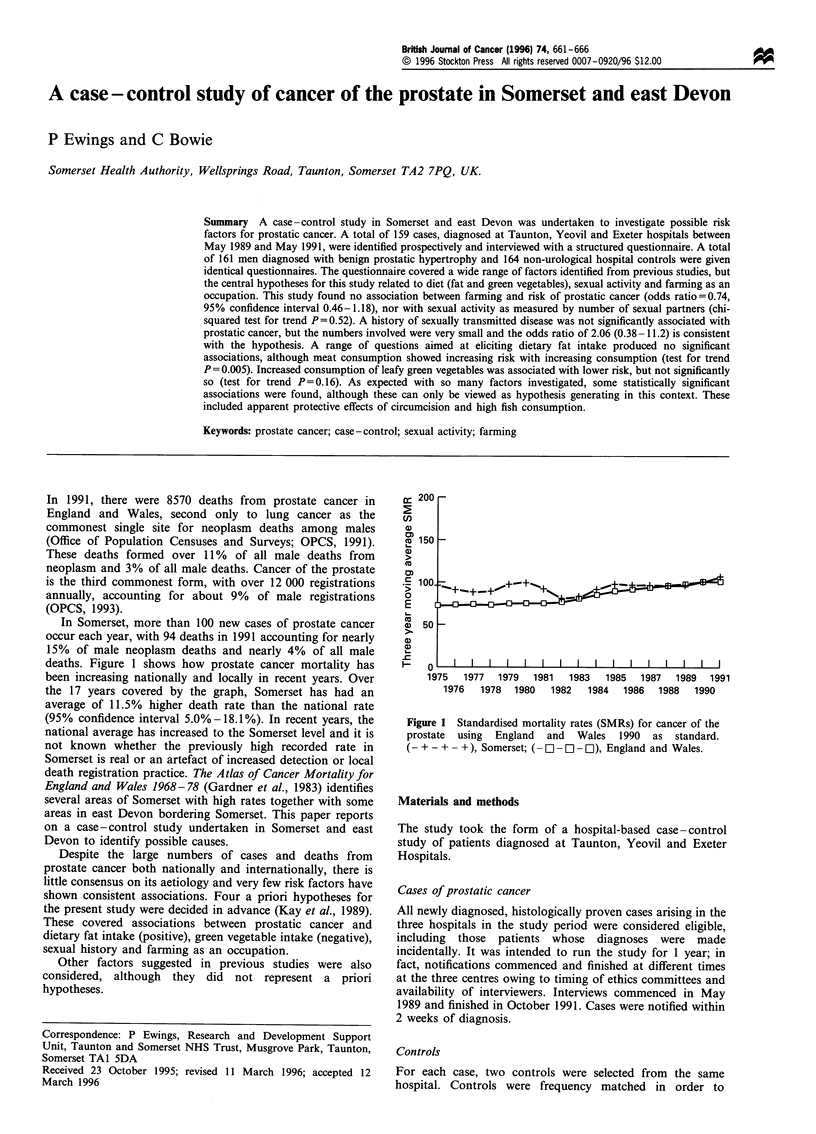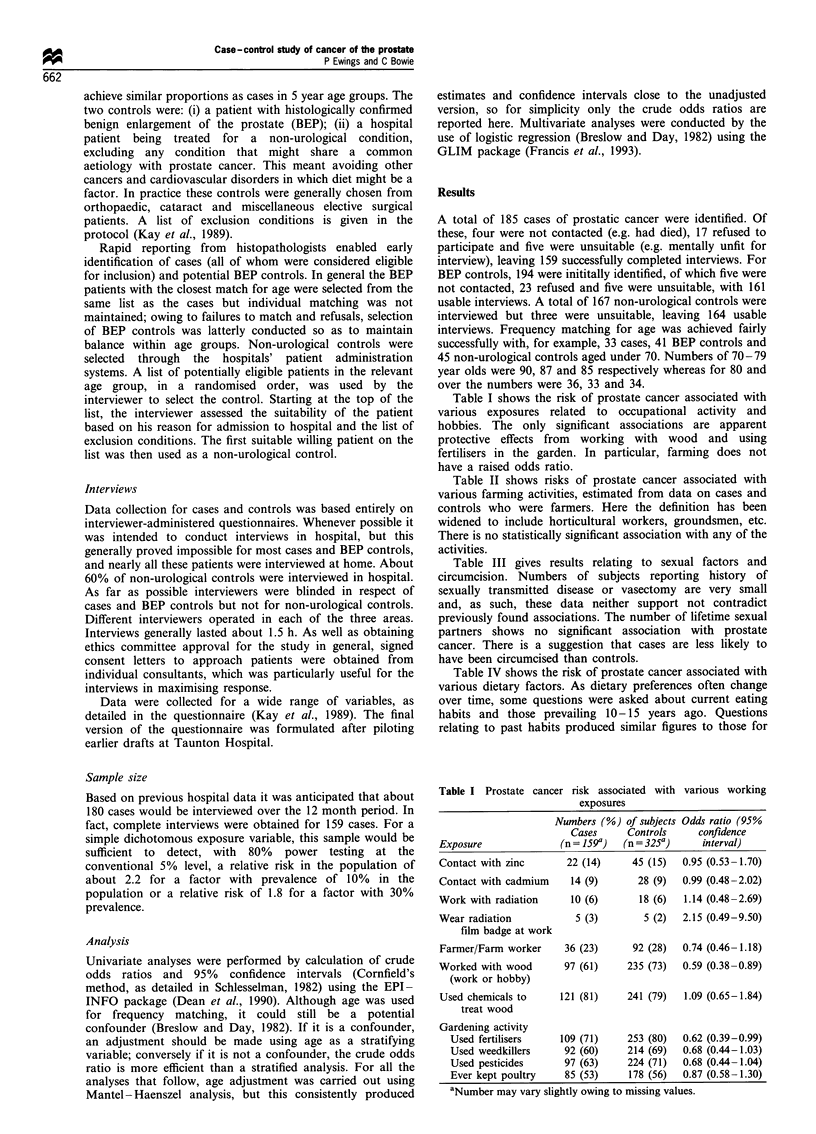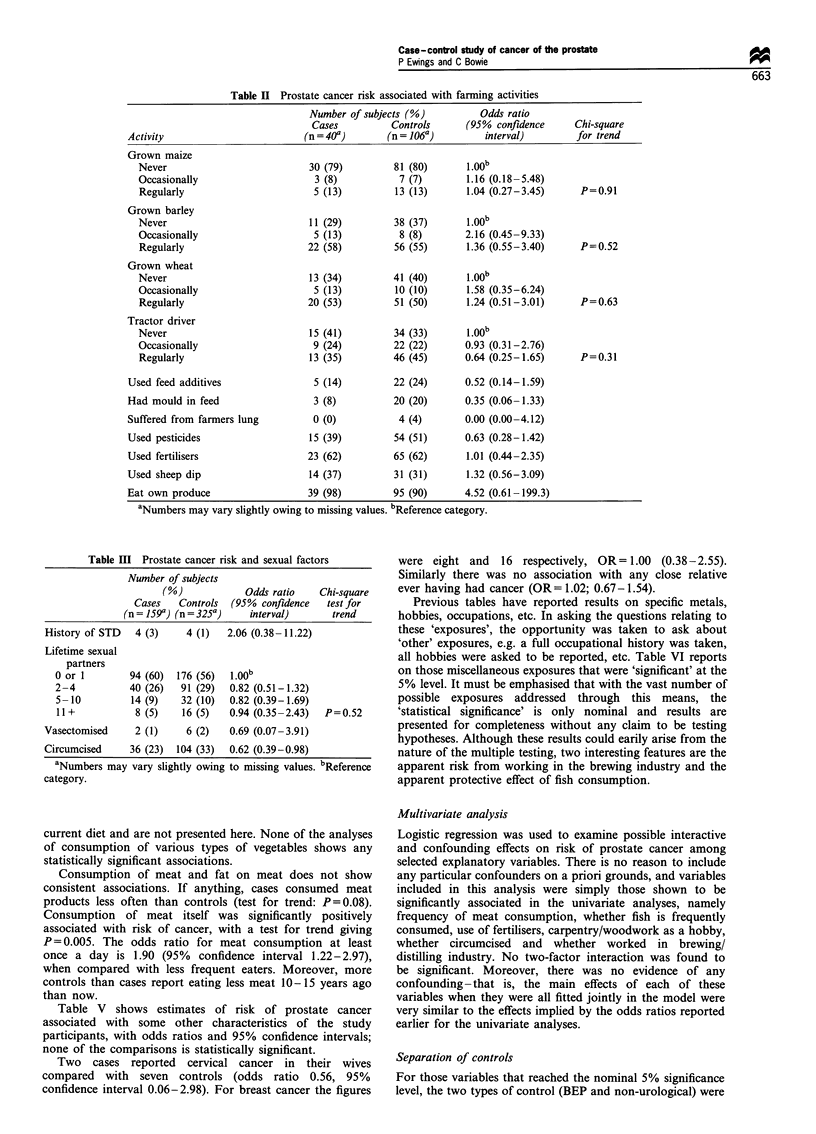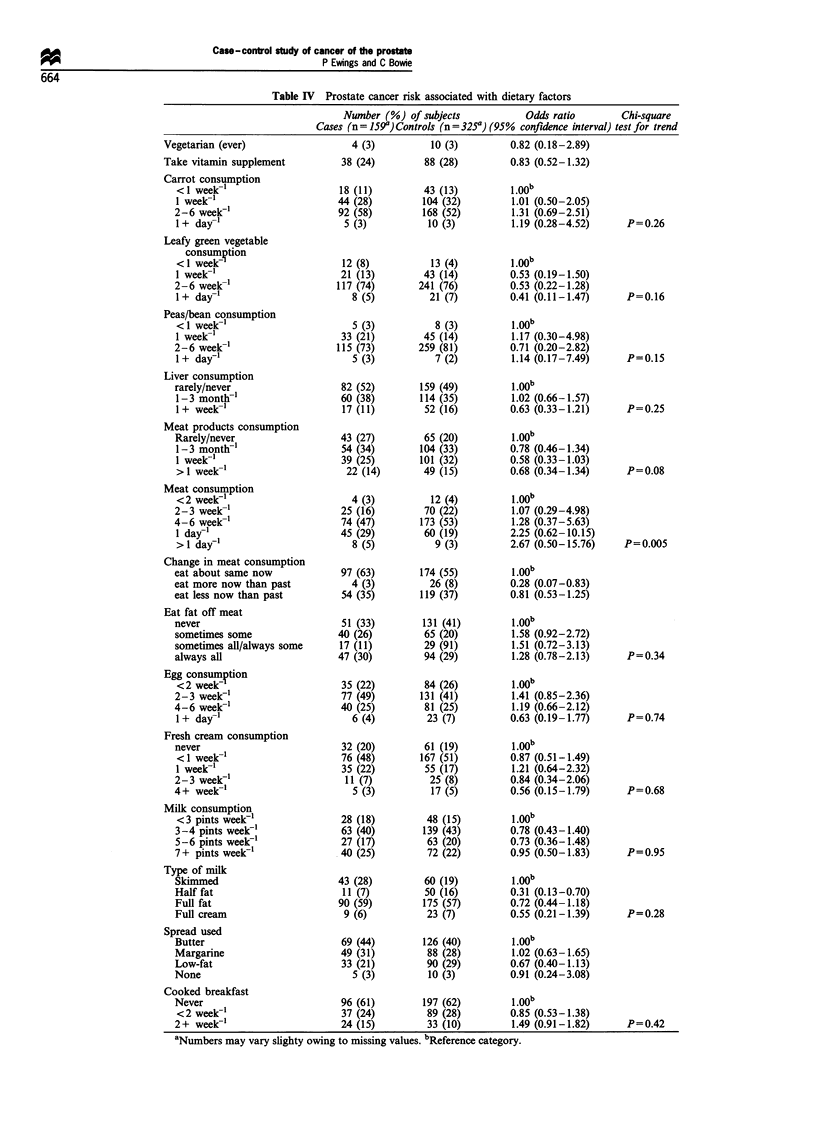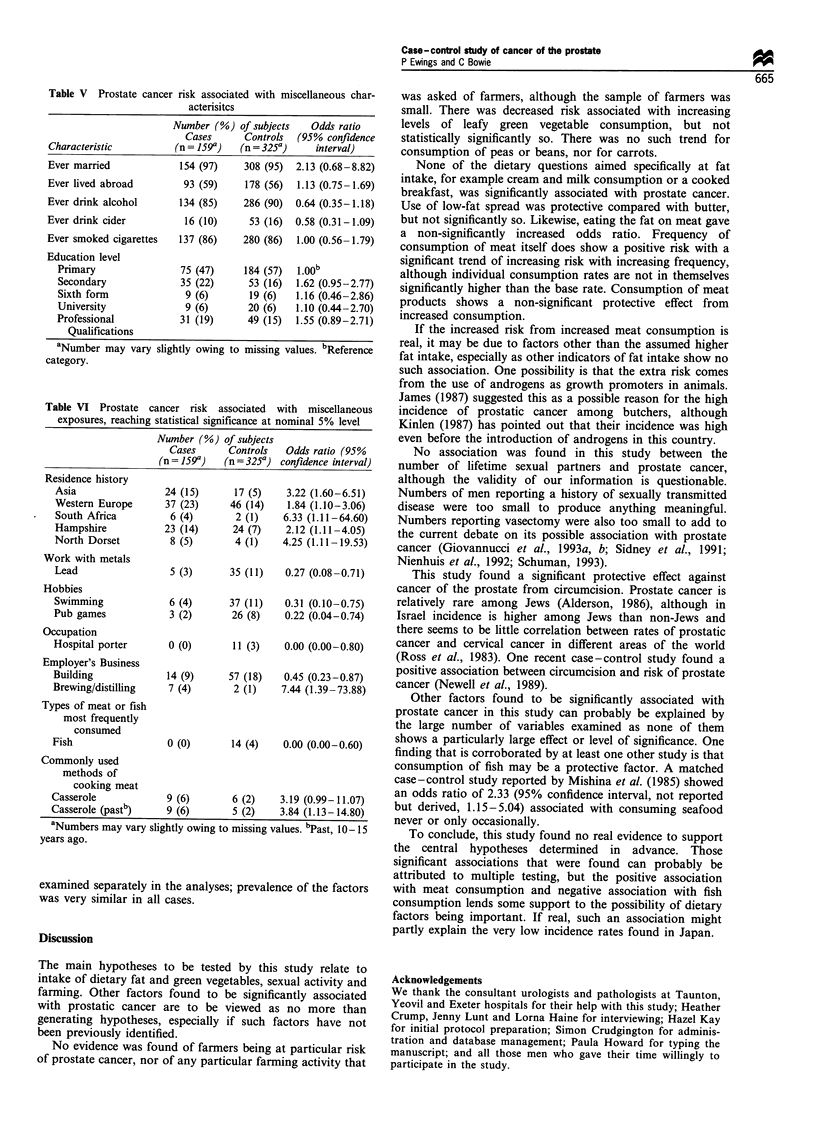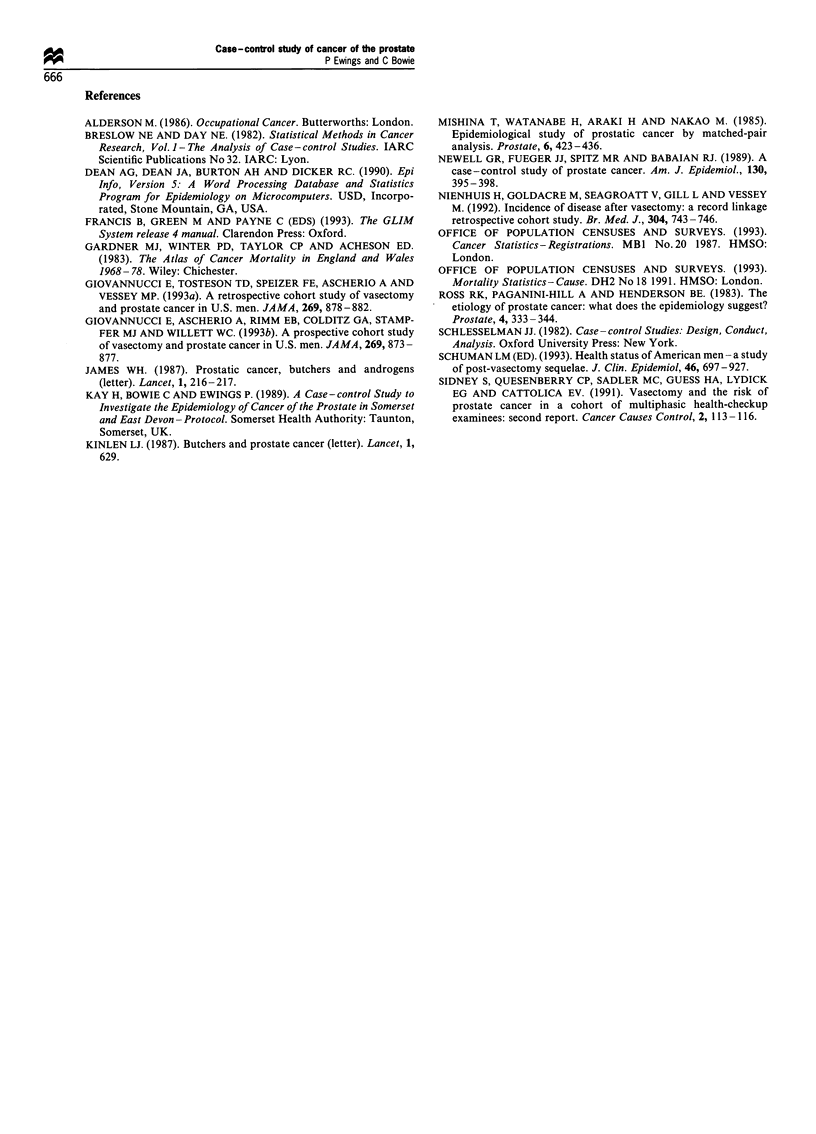# A case-control study of cancer of the prostate in Somerset and east Devon.

**DOI:** 10.1038/bjc.1996.418

**Published:** 1996-08

**Authors:** P. Ewings, C. Bowie

**Affiliations:** Somerset Health Authority, Taunton, UK.

## Abstract

A case-control study in Somerset and east Devon was undertaken to investigate possible risk factors for prostatic cancer. A total of 159 cases, diagnosed at Taunton. Yeovil and Exeter hospitals between May 1989 and May 1991, were identified prospectively and interviewed with a structured questionnaire. A total of 161 men diagnosed with benign prostatic hypertrophy and 164 non-urological hospital controls were given identical questionnaires. The questionnaire covered a wide range of factors identified from previous studies, but the central hypotheses for this study related to diet (fat and green vegetables), sexual activity and farming as an occupation. This study found no association between farming and risk of prostatic cancer (odds ratio = 0.74, 95% confidence interval 0.46-1.18), nor with sexual activity as measured by number of sexual partners (chi-squared test for trend P = 0.52). A history of sexually transmitted disease was not significantly associated with prostatic cancer, but the numbers involved were very small and the odds ratio of 2.06 (0.38-11.2) is consistent with the hypothesis. A range of questions aimed at eliciting dietary fat intake produced no significant associations, although meat consumption showed increasing risk with increasing consumption (test for trend P = 0.005). Increased consumption of leafy green vegetables was associated with lower risk, but not significantly so (test for trend P = 0.16). As expected with so many factors investigated, some statistically significant associations were found, although these can only be viewed as hypothesis generating in this context. These included apparent protective effects of circumcision and high fish consumption.


British Journal of Cancer (1996) 74, 661-666
© 1996 Stockton Press All rights reserved 0007-0920/96 $12.00

### A case-control study of cancer of the prostate in Somerset and east Devon

P Ewings and C Bowie

*Somerset Health Authority, Wellsprings Road, Taunton, Somerset TA2 7PQ, UK.*

**Summary** A case-control study in Somerset and east Devon was undertaken to investigate possible risk factors for prostatic cancer. A total of 159 cases, diagnosed at Taunton, Yeovil and Exeter hospitals between May 1989 and May 1991, were identified prospectively and interviewed with a structured questionnaire. A total of 161 men diagnosed with benign prostatic hypertrophy and 164 non-urological hospital controls were given identical questionnaires. The questionnaire covered a wide range of factors identified from previous studies, but the central hypotheses for this study related to diet (fat and green vegetables), sexual activity and farming as an occupation. This study found no association between farming and risk of prostatic cancer (odds ratio = 0.74, 95% confidence interval 0.46-1.18), nor with sexual activity as measured by number of sexual partners (chi-squared test for trend $P = 0.52$). A history of sexually transmitted disease was not significantly associated with prostatic cancer, but the numbers involved were very small and the odds ratio of 2.06 (0.38-11.2) is consistent with the hypothesis. A range of questions aimed at eliciting dietary fat intake produced no significant associations, although meat consumption showed increasing risk with increasing consumption (test for trend $P = 0.005$). Increased consumption of leafy green vegetables was associated with lower risk, but not significantly so (test for trend $P = 0.16$). As expected with so many factors investigated, some statistically significant associations were found, although these can only be viewed as hypothesis generating in this context. These included apparent protective effects of circumcision and high fish consumption.

**Keywords:** prostate cancer; case-control; sexual activity; farming

In 1991, there were 8570 deaths from prostate cancer in England and Wales, second only to lung cancer as the commonest single site for neoplasm deaths among males (Office of Population Censuses and Surveys; OPCS, 1991). These deaths formed over 11% of all male deaths from neoplasm and 3% of all male deaths. Cancer of the prostate is the third commonest form, with over 12 000 registrations annually, accounting for about 9% of male registrations (OPCS, 1993).

In Somerset, more than 100 new cases of prostate cancer occur each year, with 94 deaths in 1991 accounting for nearly 15% of male neoplasm deaths and nearly 4% of all male deaths. Figure 1 shows how prostate cancer mortality has been increasing nationally and locally in recent years. Over the 17 years covered by the graph, Somerset has had an average of 11.5% higher death rate than the national rate (95% confidence interval 5.0%-18.1%). In recent years, the national average has increased to the Somerset level and it is not known whether the previously high recorded rate in Somerset is real or an artefact of increased detection or local death registration practice. *The Atlas of Cancer Mortality for England and Wales 1968-78* (Gardner *et al.*, 1983) identifies several areas of Somerset with high rates together with some areas in east Devon bordering Somerset. This paper reports on a case-control study undertaken in Somerset and east Devon to identify possible causes.

Despite the large numbers of cases and deaths from prostate cancer both nationally and internationally, there is little consensus on its aetiology and very few risk factors have shown consistent associations. Four a priori hypotheses for the present study were decided in advance (Kay *et al.*, 1989). These covered associations between prostatic cancer and dietary fat intake (positive), green vegetable intake (negative), sexual history and farming as an occupation.

Other factors suggested in previous studies were also considered, although they did not represent a priori hypotheses.

Correspondence: P Ewings, Research and Development Support Unit, Taunton and Somerset NHS Trust, Musgrove Park, Taunton, Somerset TA1 5DA
Received 23 October 1995; revised 11 March 1996; accepted 12 March 1996

**Figure 1** Standardised mortality rates (SMRs) for cancer of the prostate using England and Wales 1990 as standard. (- + - + - +), Somerset; (-□-□-□), England and Wales.

### Materials and methods

The study took the form of a hospital-based case-control study of patients diagnosed at Taunton, Yeovil and Exeter Hospitals.

### *Cases of prostatic cancer*

All newly diagnosed, histologically proven cases arising in the three hospitals in the study period were considered eligible, including those patients whose diagnoses were made incidentally. It was intended to run the study for 1 year; in fact, notifications commenced and finished at different times at the three centres owing to timing of ethics committees and availability of interviewers. Interviews commenced in May 1989 and finished in October 1991. Cases were notified within 2 weeks of diagnosis.

### *Controls*

For each case, two controls were selected from the same hospital. Controls were frequency matched in order to achieve similar proportions as cases in 5 year age groups. The two controls were: (i) a patient with histologically confirmed benign enlargement of the prostate (BEP); (ii) a hospital patient being treated for a non-urological condition, excluding any condition that might share a common aetiology with prostate cancer. This meant avoiding other cancers and cardiovascular disorders in which diet might be a factor. In practice these controls were generally chosen from orthopaedic, cataract and miscellaneous elective surgical patients. A list of exclusion conditions is given in the protocol (Kay *et al.*, 1989).

Rapid reporting from histopathologists enabled early identification of cases (all of whom were considered eligible for inclusion) and potential BEP controls. In general the BEP patients with the closest match for age were selected from the same list as the cases but individual matching was not maintained; owing to failures to match and refusals, selection of BEP controls was latterly conducted so as to maintain balance within age groups. Non-urological controls were selected through the hospitals' patient administration systems. A list of potentially eligible patients in the relevant age group, in a randomised order, was used by the interviewer to select the control. Starting at the top of the list, the interviewer assessed the suitability of the patient based on his reason for admission to hospital and the list of exclusion conditions. The first suitable willing patient on the list was then used as a non-urological control.

*Interviews*

Data collection for cases and controls was based entirely on interviewer-administered questionnaires. Whenever possible it was intended to conduct interviews in hospital, but this generally proved impossible for most cases and BEP controls, and nearly all these patients were interviewed at home. About 60% of non-urological controls were interviewed in hospital. As far as possible interviewers were blinded in respect of cases and BEP controls but not for non-urological controls. Different interviewers operated in each of the three areas. Interviews generally lasted about 1.5 h. As well as obtaining ethics committee approval for the study in general, signed consent letters to approach patients were obtained from individual consultants, which was particularly useful for the interviews in maximising response.

Data were collected for a wide range of variables, as detailed in the questionnaire (Kay *et al.*, 1989). The final version of the questionnaire was formulated after piloting earlier drafts at Taunton Hospital.

*Sample size*

Based on previous hospital data it was anticipated that about 180 cases would be interviewed over the 12 month period. In fact, complete interviews were obtained for 159 cases. For a simple dichotomous exposure variable, this sample would be sufficient to detect, with 80% power testing at the conventional 5% level, a relative risk in the population of about 2.2 for a factor with prevalence of 10% in the population or a relative risk of 1.8 for a factor with 30% prevalence.

*Analysis*

Univariate analyses were performed by calculation of crude odds ratios and 95% confidence intervals (Cornfield's method, as detailed in Schlesselman, 1982) using the EPI-INFO package (Dean *et al.*, 1990). Although age was used for frequency matching, it could still be a potential confounder (Breslow and Day, 1982). If it is a confounder, an adjustment should be made using age as a stratifying variable; conversely if it is not a confounder, the crude odds ratio is more efficient than a stratified analysis. For all the analyses that follow, age adjustment was carried out using Mantel–Haenszel analysis, but this consistently produced estimates and confidence intervals close to the unadjusted version, so for simplicity only the crude odds ratios are reported here. Multivariate analyses were conducted by the use of logistic regression (Breslow and Day, 1982) using the GLIM package (Francis *et al.*, 1993).

### Results

A total of 185 cases of prostatic cancer were identified. Of these, four were not contacted (e.g. had died), 17 refused to participate and five were unsuitable (e.g. mentally unfit for interview), leaving 159 successfully completed interviews. For BEP controls, 194 were initially identified, of which five were not contacted, 23 refused and five were unsuitable, with 161 usable interviews. A total of 167 non-urological controls were interviewed but three were unsuitable, leaving 164 usable interviews. Frequency matching for age was achieved fairly successfully with, for example, 33 cases, 41 BEP controls and 45 non-urological controls aged under 70. Numbers of 70–79 year olds were 90, 87 and 85 respectively whereas for 80 and over the numbers were 36, 33 and 34.

Table I shows the risk of prostate cancer associated with various exposures related to occupational activity and hobbies. The only significant associations are apparent protective effects from working with wood and using fertilisers in the garden. In particular, farming does not have a raised odds ratio.

Table II shows risks of prostate cancer associated with various farming activities, estimated from data on cases and controls who were farmers. Here the definition has been widened to include horticultural workers, groundsmen, etc. There is no statistically significant association with any of the activities.

Table III gives results relating to sexual factors and circumcision. Numbers of subjects reporting history of sexually transmitted disease or vasectomy are very small and, as such, these data neither support not contradict previously found associations. The number of lifetime sexual partners shows no significant association with prostate cancer. There is a suggestion that cases are less likely to have been circumcised than controls.

Table IV shows the risk of prostate cancer associated with various dietary factors. As dietary preferences often change over time, some questions were asked about current eating habits and those prevailing 10–15 years ago. Questions relating to past habits produced similar figures to those for

**Table I** Prostate cancer risk associated with various working exposures

| *Exposure* | *Numbers (%) of subjects* *Cases* *(n = 159[a])* | *Controls* *(n = 325[a])* | *Odds ratio (95% confidence interval)* |
|---|---|---|---|
| Contact with zinc | 22 (14) | 45 (15) | 0.95 (0.53–1.70) |
| Contact with cadmium | 14 (9) | 28 (9) | 0.99 (0.48–2.02) |
| Work with radiation | 10 (6) | 18 (6) | 1.14 (0.48–2.69) |
| Wear radiation film badge at work | 5 (3) | 5 (2) | 2.15 (0.49–9.50) |
| Farmer/Farm worker | 36 (23) | 92 (28) | 0.74 (0.46–1.18) |
| Worked with wood (work or hobby) | 97 (61) | 235 (73) | 0.59 (0.38–0.89) |
| Used chemicals to treat wood | 121 (81) | 241 (79) | 1.09 (0.65–1.84) |
| Gardening activity | | | |
| Used fertilisers | 109 (71) | 253 (80) | 0.62 (0.39–0.99) |
| Used weedkillers | 92 (60) | 214 (69) | 0.68 (0.44–1.03) |
| Used pesticides | 97 (63) | 224 (71) | 0.68 (0.44–1.04) |
| Ever kept poultry | 85 (53) | 178 (56) | 0.87 (0.58–1.30) |

[a]Number may vary slightly owing to missing values.

**Table II** Prostate cancer risk associated with farming activities

| *Activity* | *Number of subjects (%)* *Cases* (n=*40*[a]) | *Controls* (n=*106*[a]) | *Odds ratio (95% confidence interval)* | *Chi-square for trend* |
|---|---|---|---|---|
| Grown maize | | | | |
| Never | 30 (79) | 81 (80) | 1.00[b] | |
| Occasionally | 3 (8) | 7 (7) | 1.16 (0.18–5.48) | |
| Regularly | 5 (13) | 13 (13) | 1.04 (0.27–3.45) | $P=0.91$ |
| Grown barley | | | | |
| Never | 11 (29) | 38 (37) | 1.00[b] | |
| Occasionally | 5 (13) | 8 (8) | 2.16 (0.45–9.33) | |
| Regularly | 22 (58) | 56 (55) | 1.36 (0.55–3.40) | $P=0.52$ |
| Grown wheat | | | | |
| Never | 13 (34) | 41 (40) | 1.00[b] | |
| Occasionally | 5 (13) | 10 (10) | 1.58 (0.35–6.24) | |
| Regularly | 20 (53) | 51 (50) | 1.24 (0.51–3.01) | $P=0.63$ |
| Tractor driver | | | | |
| Never | 15 (41) | 34 (33) | 1.00[b] | |
| Occasionally | 9 (24) | 22 (22) | 0.93 (0.31–2.76) | |
| Regularly | 13 (35) | 46 (45) | 0.64 (0.25–1.65) | $P=0.31$ |
| Used feed additives | 5 (14) | 22 (24) | 0.52 (0.14–1.59) | |
| Had mould in feed | 3 (8) | 20 (20) | 0.35 (0.06–1.33) | |
| Suffered from farmers lung | 0 (0) | 4 (4) | 0.00 (0.00–4.12) | |
| Used pesticides | 15 (39) | 54 (51) | 0.63 (0.28–1.42) | |
| Used fertilisers | 23 (62) | 65 (62) | 1.01 (0.44–2.35) | |
| Used sheep dip | 14 (37) | 31 (31) | 1.32 (0.56–3.09) | |
| Eat own produce | 39 (98) | 95 (90) | 4.52 (0.61–199.3) | |

[a]Numbers may vary slightly owing to missing values. [b]Reference category.

**Table III** Prostate cancer risk and sexual factors

| | *Number of subjects (%)* *Cases* (n=*159*[a]) | *Controls* (n=*325*[a]) | *Odds ratio (95% confidence interval)* | *Chi-square test for trend* |
|---|---|---|---|---|
| History of STD | 4 (3) | 4 (1) | 2.06 (0.38–11.22) | |
| Lifetime sexual partners | | | | |
| 0 or 1 | 94 (60) | 176 (56) | 1.00[b] | |
| 2–4 | 40 (26) | 91 (29) | 0.82 (0.51–1.32) | |
| 5–10 | 14 (9) | 32 (10) | 0.82 (0.39–1.69) | |
| 11+ | 8 (5) | 16 (5) | 0.94 (0.35–2.43) | $P=0.52$ |
| Vasectomised | 2 (1) | 6 (2) | 0.69 (0.07–3.91) | |
| Circumcised | 36 (23) | 104 (33) | 0.62 (0.39–0.98) | |

[a]Numbers may vary slightly owing to missing values. [b]Reference category.

current diet and are not presented here. None of the analyses of consumption of various types of vegetables shows any statistically significant associations.

Consumption of meat and fat on meat does not show consistent associations. If anything, cases consumed meat products less often than controls (test for trend: $P=0.08$). Consumption of meat itself was significantly positively associated with risk of cancer, with a test for trend giving $P=0.005$. The odds ratio for meat consumption at least once a day is 1.90 (95% confidence interval 1.22–2.97), when compared with less frequent eaters. Moreover, more controls than cases report eating less meat 10–15 years ago than now.

Table V shows estimates of risk of prostate cancer associated with some other characteristics of the study participants, with odds ratios and 95% confidence intervals; none of the comparisons is statistically significant.

Two cases reported cervical cancer in their wives compared with seven controls (odds ratio 0.56, 95% confidence interval 0.06–2.98). For breast cancer the figures were eight and 16 respectively, OR = 1.00 (0.38–2.55). Similarly there was no association with any close relative ever having had cancer (OR = 1.02; 0.67–1.54).

Previous tables have reported results on specific metals, hobbies, occupations, etc. In asking the questions relating to these 'exposures', the opportunity was taken to ask about 'other' exposures, e.g. a full occupational history was taken, all hobbies were asked to be reported, etc. Table VI reports on those miscellaneous exposures that were 'significant' at the 5% level. It must be emphasised that with the vast number of possible exposures addressed through this means, the 'statistical significance' is only nominal and results are presented for completeness without any claim to be testing hypotheses. Although these results could earily arise from the nature of the multiple testing, two interesting features are the apparent risk from working in the brewing industry and the apparent protective effect of fish consumption.

*Multivariate analysis*

Logistic regression was used to examine possible interactive and confounding effects on risk of prostate cancer among selected explanatory variables. There is no reason to include any particular confounders on a priori grounds, and variables included in this analysis were simply those shown to be significantly associated in the univariate analyses, namely frequency of meat consumption, whether fish is frequently consumed, use of fertilisers, carpentry/woodwork as a hobby, whether circumcised and whether worked in brewing/distilling industry. No two-factor interaction was found to be significant. Moreover, there was no evidence of any confounding–that is, the main effects of each of these variables when they were all fitted jointly in the model were very similar to the effects implied by the odds ratios reported earlier for the univariate analyses.

*Separation of controls*

For those variables that reached the nominal 5% significance level, the two types of control (BEP and non-urological) were

Table IV Prostate cancer risk associated with dietary factors

| | Number (%) of subjects Cases (n=159[a]) | Controls (n=325[a]) | Odds ratio (95% confidence interval) | Chi-square test for trend |
|---|---|---|---|---|
| Vegetarian (ever) | 4 (3) | 10 (3) | 0.82 (0.18–2.89) | |
| Take vitamin supplement | 38 (24) | 88 (28) | 0.83 (0.52–1.32) | |
| Carrot consumption | | | | |
| <1 week$^{-1}$ | 18 (11) | 43 (13) | 1.00[b] | |
| 1 week$^{-1}$ | 44 (28) | 104 (32) | 1.01 (0.50–2.05) | |
| 2–6 week$^{-1}$ | 92 (58) | 168 (52) | 1.31 (0.69–2.51) | |
| 1+ day$^{-1}$ | 5 (3) | 10 (3) | 1.19 (0.28–4.52) | $P=0.26$ |
| Leafy green vegetable consumption | | | | |
| <1 week$^{-1}$ | 12 (8) | 13 (4) | 1.00[b] | |
| 1 week$^{-1}$ | 21 (13) | 43 (14) | 0.53 (0.19–1.50) | |
| 2–6 week$^{-1}$ | 117 (74) | 241 (76) | 0.53 (0.22–1.28) | |
| 1+ day$^{-1}$ | 8 (5) | 21 (7) | 0.41 (0.11–1.47) | $P=0.16$ |
| Peas/bean consumption | | | | |
| <1 week$^{-1}$ | 5 (3) | 8 (3) | 1.00[b] | |
| 1 week$^{-1}$ | 33 (21) | 45 (14) | 1.17 (0.30–4.98) | |
| 2–6 week$^{-1}$ | 115 (73) | 259 (81) | 0.71 (0.20–2.82) | |
| 1+ day$^{-1}$ | 5 (3) | 7 (2) | 1.14 (0.17–7.49) | $P=0.15$ |
| Liver consumption | | | | |
| rarely/never | 82 (52) | 159 (49) | 1.00[b] | |
| 1–3 month$^{-1}$ | 60 (38) | 114 (35) | 1.02 (0.66–1.57) | |
| 1+ week$^{-1}$ | 17 (11) | 52 (16) | 0.63 (0.33–1.21) | $P=0.25$ |
| Meat products consumption | | | | |
| Rarely/never | 43 (27) | 65 (20) | 1.00[b] | |
| 1–3 month$^{-1}$ | 54 (34) | 104 (33) | 0.78 (0.46–1.34) | |
| 1 week$^{-1}$ | 39 (25) | 101 (32) | 0.58 (0.33–1.03) | |
| >1 week$^{-1}$ | 22 (14) | 49 (15) | 0.68 (0.34–1.34) | $P=0.08$ |
| Meat consumption | | | | |
| <2 week$^{-1}$ | 4 (3) | 12 (4) | 1.00[b] | |
| 2–3 week$^{-1}$ | 25 (16) | 70 (22) | 1.07 (0.29–4.98) | |
| 4–6 week$^{-1}$ | 74 (47) | 173 (53) | 1.28 (0.37–5.63) | |
| 1 day$^{-1}$ | 45 (29) | 60 (19) | 2.25 (0.62–10.15) | |
| >1 day$^{-1}$ | 8 (5) | 9 (3) | 2.67 (0.50–15.76) | $P=0.005$ |
| Change in meat consumption | | | | |
| eat about same now | 97 (63) | 174 (55) | 1.00[b] | |
| eat more now than past | 4 (3) | 26 (8) | 0.28 (0.07–0.83) | |
| eat less now than past | 54 (35) | 119 (37) | 0.81 (0.53–1.25) | |
| Eat fat off meat | | | | |
| never | 51 (33) | 131 (41) | 1.00[b] | |
| sometimes some | 40 (26) | 65 (20) | 1.58 (0.92–2.72) | |
| sometimes all/always some | 17 (11) | 29 (91) | 1.51 (0.72–3.13) | |
| always all | 47 (30) | 94 (29) | 1.28 (0.78–2.13) | $P=0.34$ |
| Egg consumption | | | | |
| <2 week$^{-1}$ | 35 (22) | 84 (26) | 1.00[b] | |
| 2–3 week$^{-1}$ | 77 (49) | 131 (41) | 1.41 (0.85–2.36) | |
| 4–6 week$^{-1}$ | 40 (25) | 81 (25) | 1.19 (0.66–2.12) | |
| 1+ day$^{-1}$ | 6 (4) | 23 (7) | 0.63 (0.19–1.77) | $P=0.74$ |
| Fresh cream consumption | | | | |
| never | 32 (20) | 61 (19) | 1.00[b] | |
| <1 week$^{-1}$ | 76 (48) | 167 (51) | 0.87 (0.51–1.49) | |
| 1 week$^{-1}$ | 35 (22) | 55 (17) | 1.21 (0.64–2.32) | |
| 2–3 week$^{-1}$ | 11 (7) | 25 (8) | 0.84 (0.34–2.06) | |
| 4+ week$^{-1}$ | 5 (3) | 17 (5) | 0.56 (0.15–1.79) | $P=0.68$ |
| Milk consumption | | | | |
| <3 pints week$^{-1}$ | 28 (18) | 48 (15) | 1.00[b] | |
| 3–4 pints week$^{-1}$ | 63 (40) | 139 (43) | 0.78 (0.43–1.40) | |
| 5–6 pints week$^{-1}$ | 27 (17) | 63 (20) | 0.73 (0.36–1.48) | |
| 7+ pints week$^{-1}$ | 40 (25) | 72 (22) | 0.95 (0.50–1.83) | $P=0.95$ |
| Type of milk | | | | |
| Skimmed | 43 (28) | 60 (19) | 1.00[b] | |
| Half fat | 11 (7) | 50 (16) | 0.31 (0.13–0.70) | |
| Full fat | 90 (59) | 175 (57) | 0.72 (0.44–1.18) | |
| Full cream | 9 (6) | 23 (7) | 0.55 (0.21–1.39) | $P=0.28$ |
| Spread used | | | | |
| Butter | 69 (44) | 126 (40) | 1.00[b] | |
| Margarine | 49 (31) | 88 (28) | 1.02 (0.63–1.65) | |
| Low-fat | 33 (21) | 90 (29) | 0.67 (0.40–1.13) | |
| None | 5 (3) | 10 (3) | 0.91 (0.24–3.08) | |
| Cooked breakfast | | | | |
| Never | 96 (61) | 197 (62) | 1.00[b] | |
| <2 week$^{-1}$ | 37 (24) | 89 (28) | 0.85 (0.53–1.38) | |
| 2+ week$^{-1}$ | 24 (15) | 33 (10) | 1.49 (0.91–1.82) | $P=0.42$ |

[a]Numbers may vary slighty owing to missing values. [b]Reference category.

**Table V** Prostate cancer risk associated with miscellaneous characterisitcs

| *Characteristic* | *Number (%) of subjects* Cases (n = 159[a]) | Controls (n = 325[a]) | *Odds ratio (95% confidence interval)* |
|---|---|---|---|
| Ever married | 154 (97) | 308 (95) | 2.13 (0.68-8.82) |
| Ever lived abroad | 93 (59) | 178 (56) | 1.13 (0.75-1.69) |
| Ever drink alcohol | 134 (85) | 286 (90) | 0.64 (0.35-1.18) |
| Ever drink cider | 16 (10) | 53 (16) | 0.58 (0.31-1.09) |
| Ever smoked cigarettes | 137 (86) | 280 (86) | 1.00 (0.56-1.79) |
| Education level | | | |
| Primary | 75 (47) | 184 (57) | 1.00[b] |
| Secondary | 35 (22) | 53 (16) | 1.62 (0.95-2.77) |
| Sixth form | 9 (6) | 19 (6) | 1.16 (0.46-2.86) |
| University | 9 (6) | 20 (6) | 1.10 (0.44-2.70) |
| Professional Qualifications | 31 (19) | 49 (15) | 1.55 (0.89-2.71) |

[a]Number may vary slightly owing to missing values. [b]Reference category.

**Table VI** Prostate cancer risk associated with miscellaneous exposures, reaching statistical significance at nominal 5% level

| | *Number (%) of subjects* Cases (n = 159[a]) | Controls (n = 325[a]) | *Odds ratio (95% confidence interval)* |
|---|---|---|---|
| Residence history | | | |
| Asia | 24 (15) | 17 (5) | 3.22 (1.60-6.51) |
| Western Europe | 37 (23) | 46 (14) | 1.84 (1.10-3.06) |
| South Africa | 6 (4) | 2 (1) | 6.33 (1.11-64.60) |
| Hampshire | 23 (14) | 24 (7) | 2.12 (1.11-4.05) |
| North Dorset | 8 (5) | 4 (1) | 4.25 (1.11-19.53) |
| Work with metals | | | |
| Lead | 5 (3) | 35 (11) | 0.27 (0.08-0.71) |
| Hobbies | | | |
| Swimming | 6 (4) | 37 (11) | 0.31 (0.10-0.75) |
| Pub games | 3 (2) | 26 (8) | 0.22 (0.04-0.74) |
| Occupation | | | |
| Hospital porter | 0 (0) | 11 (3) | 0.00 (0.00-0.80) |
| Employer's Business | | | |
| Building | 14 (9) | 57 (18) | 0.45 (0.23-0.87) |
| Brewing/distilling | 7 (4) | 2 (1) | 7.44 (1.39-73.88) |
| Types of meat or fish most frequently consumed | | | |
| Fish | 0 (0) | 14 (4) | 0.00 (0.00-0.60) |
| Commonly used methods of cooking meat | | | |
| Casserole | 9 (6) | 6 (2) | 3.19 (0.99-11.07) |
| Casserole (past[b]) | 9 (6) | 5 (2) | 3.84 (1.13-14.80) |

[a]Numbers may vary slightly owing to missing values. [b]Past, 10-15 years ago.

examined separately in the analyses; prevalence of the factors was very similar in all cases.

### Discussion

The main hypotheses to be tested by this study relate to intake of dietary fat and green vegetables, sexual activity and farming. Other factors found to be significantly associated with prostatic cancer are to be viewed as no more than generating hypotheses, especially if such factors have not been previously identified.

No evidence was found of farmers being at particular risk of prostate cancer, nor of any particular farming activity that was asked of farmers, although the sample of farmers was small. There was decreased risk associated with increasing levels of leafy green vegetable consumption, but not statistically significantly so. There was no such trend for consumption of peas or beans, nor for carrots.

None of the dietary questions aimed specifically at fat intake, for example cream and milk consumption or a cooked breakfast, was significantly associated with prostate cancer. Use of low-fat spread was protective compared with butter, but not significantly so. Likewise, eating the fat on meat gave a non-significantly increased odds ratio. Frequency of consumption of meat itself does show a positive risk with a significant trend of increasing risk with increasing frequency, although individual consumption rates are not in themselves significantly higher than the base rate. Consumption of meat products shows a non-significant protective effect from increased consumption.

If the increased risk from increased meat consumption is real, it may be due to factors other than the assumed higher fat intake, especially as other indicators of fat intake show no such association. One possibility is that the extra risk comes from the use of androgens as growth promoters in animals. James (1987) suggested this as a possible reason for the high incidence of prostatic cancer among butchers, although Kinlen (1987) has pointed out that their incidence was high even before the introduction of androgens in this country.

No association was found in this study between the number of lifetime sexual partners and prostate cancer, although the validity of our information is questionable. Numbers of men reporting a history of sexually transmitted disease were too small to produce anything meaningful. Numbers reporting vasectomy were also too small to add to the current debate on its possible association with prostate cancer (Giovannucci *et al.*, 1993*a*, *b*; Sidney *et al.*, 1991; Nienhuis *et al.*, 1992; Schuman, 1993).

This study found a significant protective effect against cancer of the prostate from circumcision. Prostate cancer is relatively rare among Jews (Alderson, 1986), although in Israel incidence is higher among Jews than non-Jews and there seems to be little correlation between rates of prostatic cancer and cervical cancer in different areas of the world (Ross *et al.*, 1983). One recent case-control study found a positive association between circumcision and risk of prostate cancer (Newell *et al.*, 1989).

Other factors found to be significantly associated with prostate cancer in this study can probably be explained by the large number of variables examined as none of them shows a particularly large effect or level of significance. One finding that is corroborated by at least one other study is that consumption of fish may be a protective factor. A matched case-control study reported by Mishina *et al.* (1985) showed an odds ratio of 2.33 (95% confidence interval, not reported but derived, 1.15-5.04) associated with consuming seafood never or only occasionally.

To conclude, this study found no real evidence to support the central hypotheses determined in advance. Those significant associations that were found can probably be attributed to multiple testing, but the positive association with meat consumption and negative association with fish consumption lends some support to the possibility of dietary factors being important. If real, such an association might partly explain the very low incidence rates found in Japan.

**Acknowledgements**
We thank the consultant urologists and pathologists at Taunton, Yeovil and Exeter hospitals for their help with this study; Heather Crump, Jenny Lunt and Lorna Haine for interviewing; Hazel Kay for initial protocol preparation; Simon Crudgington for administration and database management; Paula Howard for typing the manuscript; and all those men who gave their time willingly to participate in the study.

### References

ALDERSON M. (1986). *Occupational Cancer*. Butterworths: London.

BRESLOW NE AND DAY NE. (1982). *Statistical Methods in Cancer Research, Vol. 1 – The Analysis of Case–control Studies*. IARC Scientific Publications No 32. IARC: Lyon.

DEAN AG, DEAN JA, BURTON AH AND DICKER RC. (1990). *Epi Info, Version 5: A Word Processing Database and Statistics Program for Epidemiology on Microcomputers*. USD, Incorporated, Stone Mountain, GA, USA.

FRANCIS B, GREEN M AND PAYNE C (EDS) (1993). *The GLIM System release 4 manual*. Clarendon Press: Oxford.

GARDNER MJ, WINTER PD, TAYLOR CP AND ACHESON ED. (1983). *The Atlas of Cancer Mortality in England and Wales 1968–78*. Wiley: Chichester.

GIOVANNUCCI E, TOSTESON TD, SPEIZER FE, ASCHERIO A AND VESSEY MP. (1993*a*). A retrospective cohort study of vasectomy and prostate cancer in U.S. men. *JAMA*, **269**, 878–882.

GIOVANNUCCI E, ASCHERIO A, RIMM EB, COLDITZ GA, STAMPFER MJ AND WILLETT WC. (1993*b*). A prospective cohort study of vasectomy and prostate cancer in U.S. men. *JAMA*, **269**, 873–877.

JAMES WH. (1987). Prostatic cancer, butchers and androgens (letter). *Lancet*, **1**, 216–217.

KAY H, BOWIE C AND EWINGS P. (1989). *A Case–control Study to Investigate the Epidemiology of Cancer of the Prostate in Somerset and East Devon – Protocol*. Somerset Health Authority: Taunton, Somerset, UK.

KINLEN LJ. (1987). Butchers and prostate cancer (letter). *Lancet*, **1**, 629.

MISHINA T, WATANABE H, ARAKI H AND NAKAO M. (1985). Epidemiological study of prostatic cancer by matched-pair analysis. *Prostate*, **6**, 423–436.

NEWELL GR, FUEGER JJ, SPITZ MR AND BABAIAN RJ. (1989). A case–control study of prostate cancer. *Am. J. Epidemiol.*, **130**, 395–398.

NIENHUIS H, GOLDACRE M, SEAGROATT V, GILL L AND VESSEY M. (1992). Incidence of disease after vasectomy: a record linkage retrospective cohort study. *Br. Med. J.*, **304**, 743–746.

OFFICE OF POPULATION CENSUSES AND SURVEYS. (1993). *Cancer Statistics – Registrations*. MB1 No. 20 1987. HMSO: London.

OFFICE OF POPULATION CENSUSES AND SURVEYS. (1993). *Mortality Statistics – Cause*. DH2 No 18 1991. HMSO: London.

ROSS RK, PAGANINI-HILL A AND HENDERSON BE. (1983). The etiology of prostate cancer: what does the epidemiology suggest? *Prostate*, **4**, 333–344.

SCHLESSELMAN JJ. (1982). *Case–control Studies: Design, Conduct, Analysis*. Oxford University Press: New York.

SCHUMAN LM (ED). (1993). Health status of American men – a study of post-vasectomy sequelae. *J. Clin. Epidemiol*, **46**, 697–927.

SIDNEY S, QUESENBERRY CP, SADLER MC, GUESS HA, LYDICK EG AND CATTOLICA EV. (1991). Vasectomy and the risk of prostate cancer in a cohort of multiphasic health-checkup examinees: second report. *Cancer Causes Control*, **2**, 113–116.